# Primary Intestinal Extranodal Natural Killer/T-Cell Lymphoma, Nasal Type: A Comprehensive Clinicopathological Analysis of 55 Cases

**DOI:** 10.1371/journal.pone.0161831

**Published:** 2016-08-26

**Authors:** Bao-Hua Yu, Ruo-Hong Shui, Wei-Qi Sheng, Chao-Fu Wang, Hong-Fen Lu, Xiao-Yan Zhou, Xiong-Zeng Zhu, Xiao-Qiu Li

**Affiliations:** 1 Department of Pathology, Fudan University Shanghai Cancer Center, Shanghai, China; 2 Department of Oncology, Shanghai Medical College, Fudan University, Shanghai, China; Gustave Roussy, FRANCE

## Abstract

**Purpose:**

To investigate the clinicopathological features, survival and prognostic factors of primary intestinal extranodal natural killer/T-cell lymphoma, nasal type (PI-ENKTCL).

**Methods:**

Clinical and histological characteristics of PI-ENKTCL cases were retrospectively evaluated. Immunohistochemical phenotype and status of Epstein-Barr virus (EBV) and T-cell receptor (TCR) gene rearrangement were examined. The overall survival and prognostic parameters were also analyzed.

**Results:**

Fifty-five (2.7%) cases with PI-ENKTCL were identified out of 2017 archived ENKTCL cases, with a median age of 39 years and a male to female ratio of 2.1:1. The most common symptom was abdominal pain (90.9%), accompanied frequently with fever and less commonly with intestinal perforation or B symptoms. Small intestine (50.9%) was the most common site to be involved. 47.3% and 36.4% cases presented with stage I and II diseases, respectively. Histologically, most cases displayed characteristic morphologic changes of ENKTCL. Cytoplasmic CD3, TIA-1 and CD56 expression was found in 100%, 94.5% and 89.1% of cases, respectively. In situ hybridization detection for EBV demonstrated positive results in all cases. Monoclonal TCR gene rearrangement was found in 52.9% of tested cases. Chemotherapy with a DICE or L-asparaginase/peg-asparginase-containing regimen was most often employed. Both advanced tumor stage and B symptoms were independent inferior prognostic factors (*p* = 0.001 and *p* = 0.010). Noticeably, 6 cases demonstrated a CD4-positive phenotype. These cases featured a relatively older median age (58 years), predominance of small/medium-sized neoplastic cells, a higher rate of TCR rearrangement and slightly favorable outcome.

**Conclusion:**

We reported by far the largest series of PI-ENKTCL, and demonstrated its heterogeneity, aggressive clinical behavior and unsatisfying response to the current therapeutic strategies. Those CD4-positive cases might represent a unique subtype of PI-ENKTCL or distinct entity. Further investigations are required for the better understanding and management of this unusual disease.

## Introduction

Extranodal natural killer (NK)/T-cell lymphoma, nasal type (ENKTCL), a rare distinct malignancy that comprises 3–8% of all lymphomas, is most prevalent in Asian and Central and South American populations [1]. This tumor predominantly involves extranodal sites, and features pathologically vascular invasion, prominent necrosis, cytotoxic phenotype and association with Epstein-Barr virus (EBV) [2, 3]. While most ENKTCL cases derived from NK cells, some show a cytotoxic T-cell phenotype [3, 4]. Approximately 80% of ENKTCL cases occur in the upper aerodigestive tract, with the nasal cavity being mostly affected [5, 6], other preferential sites of involvement include the skin, soft tissue, gastrointestinal (GI) tract and the gonad [7, 8]. Primary intestinal ENKTCL (PI-ENKTCL) is rare, which accounts for 3.1% of all intestinal non-Hodgkin lymphoma cases according to the literature [9]. Due to its rarity, the clinical and pathological features of intestinal ENKTCL have not been well illustrated, which may lead to dilemmas not only in the diagnosis but also the treatment of this disease. Furthermore, data regarding the therapeutic strategies and prognostic factors of this peculiar lymphoma is still limited. We thus retrospectively analyzed by far the largest series of PI-ENKTCL, for the purpose of better understanding the clinicopathological features of this rare tumor, which is of paramount importance for the accurate diagnosis and appropriate treatment.

## Materials and Methods

### Case selection

Altogether 55 cases with PI-ENKTCL, diagnosed between January 2007 and August 2015, were retrieved from the files of Department of Pathology, Fudan University Shanghai Cancer Center (Shanghai, China). For all cases, a primary intestinal manifestation of the disease was confirmed by a precise staging work-up through a computed tomography (CT) staging and/or PET-CT scan. And those with a probable secondary involvement of the intestine were not included. Pathological diagnosis was made by two of the authors (BHY and XQL) according to the criteria described in the WHO classification of tumors of the haematopoietic and lymphoid tissues [2]. Clinical data including the follow up information were also collected and analyzed.

### Histology and immunohistochemistry

Archival formalin-fixed paraffin-embedded tumor tissues were recut for a routine hematoxylin and eosin (H&E) stain and the immunohistochemical procedure. Histological characteristics, including the cytological details of the tumor, the presence of necrosis and ulceration, angiocentricity and angiodestruction, admixed inflammatory infiltrates, and the depth of the neoplastic infiltration, were reviewed and assessed by three of the authors (BHY, XYZ and XQL) under a multi-headed microscope.

The immunohistochemical study was performed using a Ventana Bench Mark ultra autostainer (Ventana Medical System Inc., Roche Tuson, AZ, USA) and the Ventana ultra view universal DAB detection kit. The primary antibodies against CD20, CD2, cytoplasmic CD3 [CD3 (epsilon)], CD4, CD5, CD7, CD8, CD30, CD56, T-cell-restricted intracellular antigen-1 (TIA-1), granzyme B (GrB), perforin, ALK1 and Ki-67 were employed in the present study. Except for TIA-1 (Dako, Glostrup, Denmark), all of the above-mentioned antibodies were commercial products by Roche Ventana. For each stain, a parallel stain using appropriate positive and negative controls was performed. The Ki-67 labeling index was estimated to the closest decile.

### In situ hybridization (ISH) detection for EBV-encoded small RNA (EBER)

The status of EBV infection was assessed by an ISH detection for EBER on paraffin-embedded tissue sections using fluorescein-labeled oligonucleotide probes (INFROM EBER Probe, Ventana), as previously described [10]. The visualization system used was the Bench Mark XT with enzymatic digestion (ISH Protease 2, Ventana) and the iVIEW Blue v3 detection kit (Ventana). Appropriate positive and negative control sections were included for each run.

### Polymerase chain reaction (PCR) assays for T-cell receptor (TCR) gene rearrangement

Genomic DNA was extracted from formalin-fixed paraffin-embedded tissues, using the QIAamp mini kit (Qiagen, GmbH, Germany), and the concentration of DNA was measured by a spectrophotometer. Rearrangement of TCR-β, γ, δ genes was detected by multiplex PCR assays according to standard techniques, as described previously [11]. Amplifiability of the DNA was confirmed by concurrent PCR amplification of the β-globin sequence. Each PCR study was carried out in duplicate and included positive, negative, and no-template controls. The PCR products were analyzed by capillary electrophoresis as previously documented [11], using the ABI PRISM 310 Genetic Analyzer (Applied Biosystems, CA, USA).

### Statistical analysis

Overall survival (OS) was defined as the interval from the initial diagnosis to the date of death from any cause or the last contact. The OS was estimated using the Kaplan-Meier method and was compared by means of the log-rank test. Multivariate analyses were also carried out using the Cox proportional hazard regression model to identify prognostic factors. The clinicopathologic parameters for assessment included age, tumor location, stage of disease, B symptoms [including fever (temperature >38°C) for 3 consecutive days, night sweats, and/or weight loss exceeding 10% of body weight in 6 months], intestinal perforation, size and immunophenotype of tumor cells, as well as TCR gene rearrangement status. All the statistical analyses were conducted using the SPSS software package (SPSS version 19.0; SPSS Inc., Chicago IL, USA). A *p* value of <0.05 was considered statistically significant.

### Ethics statement

This study was approved by the Institutional Review Board of Fudan University Shanghai Cancer Center (Shanghai Cancer Center Ethical Committee). The patient records/information was anonymized and de-identified prior to analysis.

## Results

### Clinical findings

Altogether 2017 ENKTCL cases were documented in our laboratory database during the period between 2007 and 2015, among which only 55 (2.7%) were proved to present with primary intestinal lesions. There were 37 male and 18 female patients, with a male-to-female ratio of 2.1:1. The average and median age at diagnosis was 43 and 39 years, respectively (range, 14–75 years). The most common symptom at diagnosis was abdominal pain (50 patients, 90.9%). Thirty-one patients (56.4%) had fever, and 10 (18.2%) presented with lower GI bleeding or fecal occult blood. Intestinal perforation and B symptoms were observed in 18 (32.7%) and 19 (34.5%) patients, respectively. Other concomitant clinical manifestations included diarrhea, nausea, vomiting, abdominal mass, intestinal obstruction and constipation.

With regard to the anatomic sites of involvement, 28 (50.9%) tumors occurred in the small intestine (including the duodenum, jejunum and ileum). Eleven (20.0%) and 13 (23.6%) were located in the ileocecal junction and colon, respectively. And the remaining 3 (5.5%) had multifocal lesions involving at least two different intestinal segments. Mesentery lymph node involvement was observed in 14 (36.8%) out of 38 cases. According to the Lugano staging system, 26 cases (47.3%) in the current series presented with stage I diseases, and 20 (36.4%) and 9 (16.4%) with stage II and advanced stage (stage III/IV) diseases, respectively.

### Histological findings

**Fig 1:**
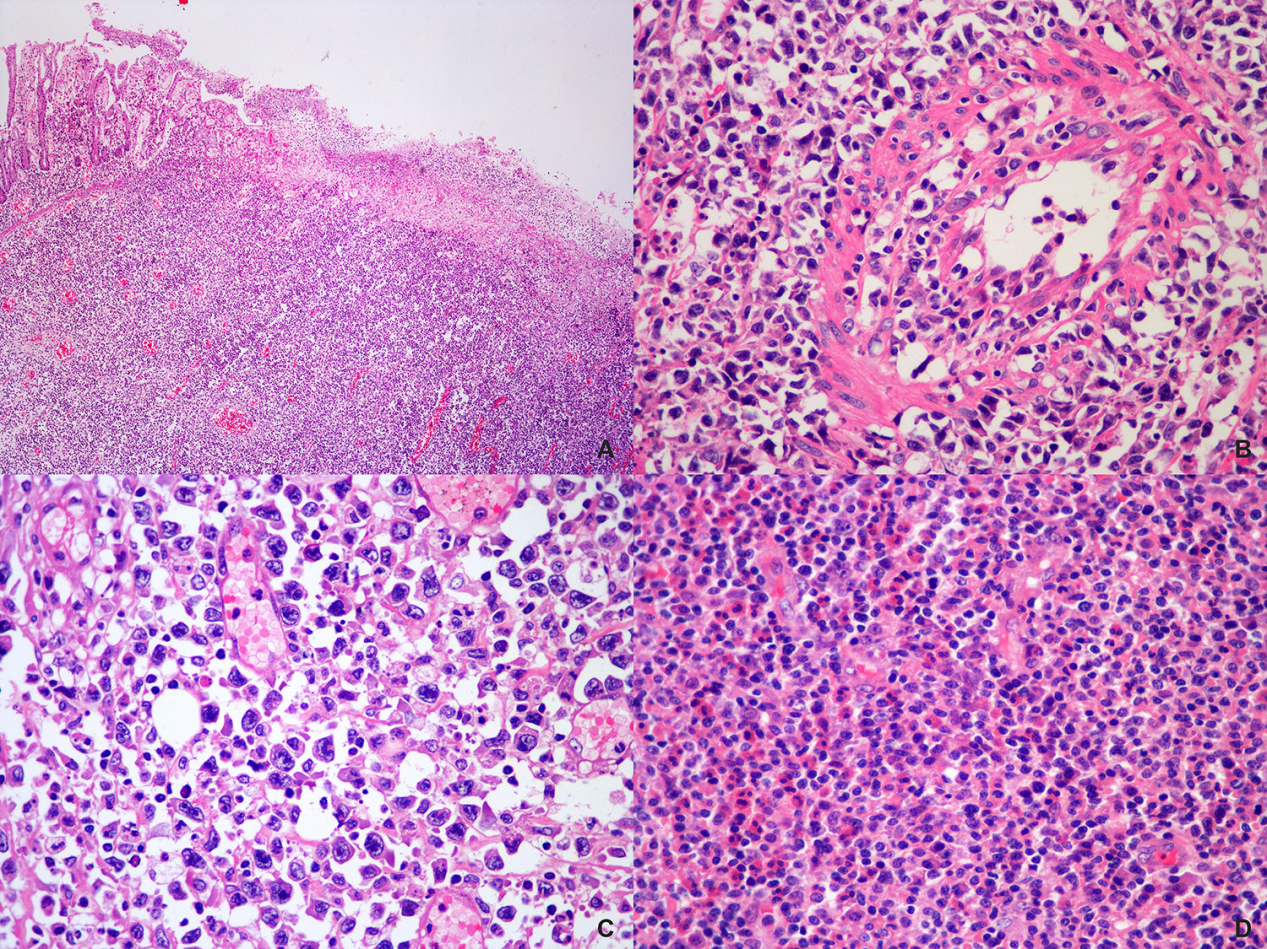
Histological features of PI-ENKTCL. (A) A low power view of the H&E stained section showed the overlying mucosa was partially effaced and the neoplastic cells extensively infiltrated the muscular wall. (B) A blood vessel with invasion by the neoplastic cells was shown. (C) A high power view demonstrated that the tumor was composed of a mixture of small, medium and large atypical lymphoid cells, with some hallmark cells identified. (D) Numerous eosinophils and plasma cells were intermingled with the tumor cells.

Overall, the specimens from the 55 cases comprised 42 resected tumors and 13 endoscopical biopsies. Ulceration and necrosis of the overlying mucosa, ranging from focal to extensive, were noticed in all cases. The atypical lymphoid cells infiltrated and effaced the mucosal architecture, which is more easily to be appreciated in the resected tumors (Fig 1A). Geographic necrosis was also frequently observed. Angiocentric /angiodestructive growth pattern of the tumor cells was another common feature noticed in these specimens (Fig 1B). Of the 42 cases with resected specimens, tumor invaded muscularis propria in 2 (4.8%), subserosa in 30 (71.4%), and penetrated the intestinal wall and spread beyond in 10 (23.8%) cases. Cytologically, the neoplastic lymphoid cells exhibited a broad spectrum in their size and appearance. Twenty-four cases (43.6%) displayed a mixed population of small, medium to large cells, 18 cases (32.7%) were composed predominantly of small to medium-sized cells, 12 cases (21.8%) predominated by medium-sized cells, and the remaining one (1.8%) was composed of uniform small cells. The small and intermediate cells often had oval or slightly irregular convoluted nuclei, hyperchromatic or granulated chromatin and inconspicuous nucleoli. The larger cells might have exceedingly irregular nuclei with vesicular or coarsely clumped chromatin and prominent nucleoli. Moderate to abundant, clear or lightly eosinophilic cytoplasm was seen in most cases. In occasional cases, the tumor cells showed a striking atypia with noticeable horseshoe- or kidney-shaped nuclei (Fig 1C). In addition, mononucleated or multinucleated giant cells with striking bizarre appearance were identified in 5 (9.1%) cases. Mitotic figures and varying amounts of apoptotic bodies were easily identified in most cases. Inflammatory infiltrates, consisting of small lymphocytes, plasma cells, histiocytes and eosinophils, were admixed with the tumor cell populations in variable proportions from case to case (Fig 1D). In one case, deposits of calcified schistosome ova were noticed in the intestinal wall and lymph nodes.

### Immunohistochemistry, ISH for EBER, and TCR gene rearrangement

**Fig 2:**
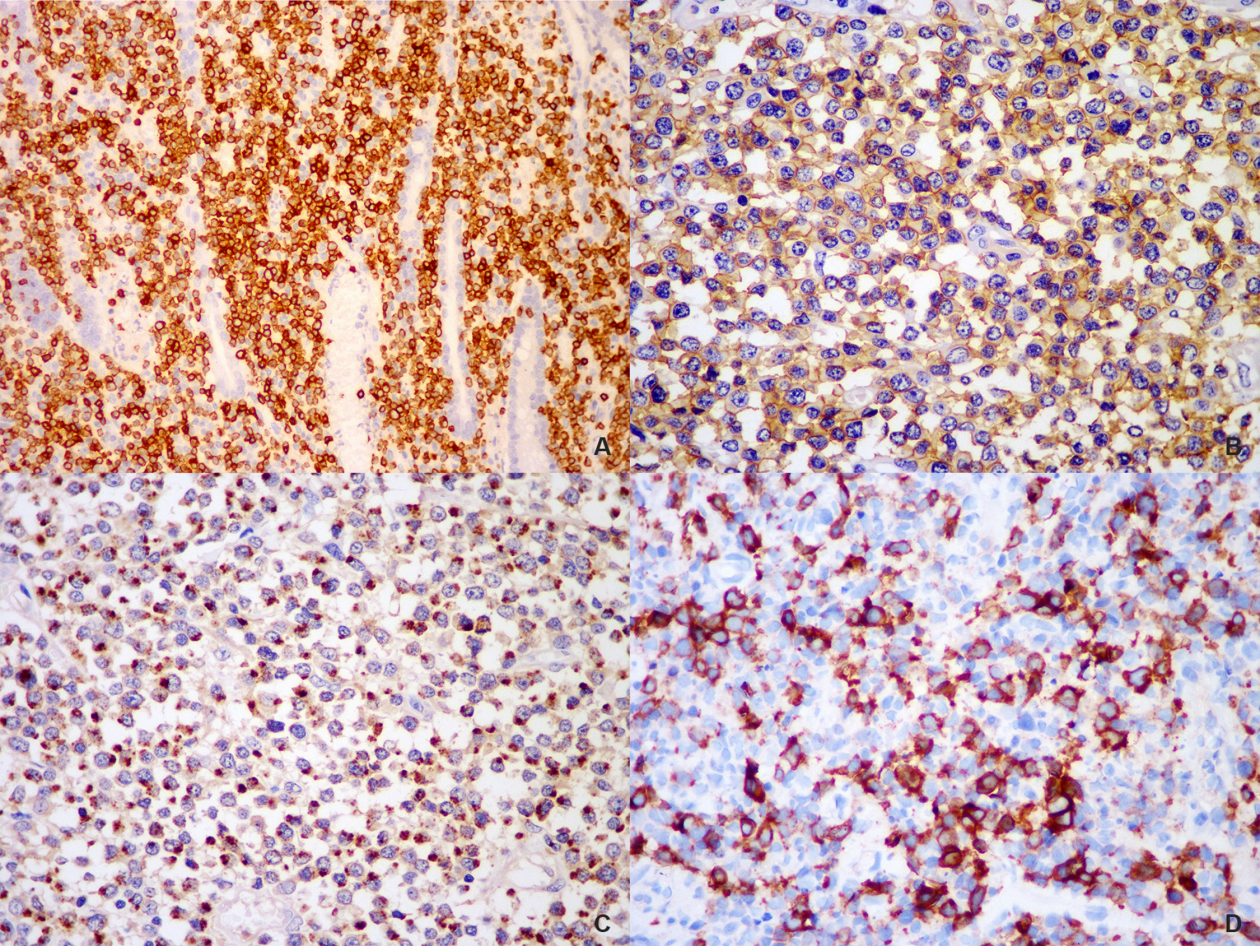
Immunophenotypic features of PI-ENKTCL. The neoplastic cells were positive for CD3 (A), CD56 (B) and TIA-1 (C). Positivity for CD30 (D) was seen in some larger atypical cells.

Immunohistochemically, tumor cells of all cases were positive, either diffusely or partially, for cytoplasmic CD3. Positivity for CD4 and CD8 of tumor cells was found in 14.0% (6/43) and 2.4% (1/41) of the tested cases, respectively, but none expressed both antigens. Other T or NK markers, such as CD2, CD5 and CD7, were expressed in a variable proportion of all cases. Positive CD56 immunostaining was observed in 89.1% (49/55) of cases. Tumor cells were consistently positive for the cytotoxic marker TIA-1 (52/55, 94.5%), less frequently, the expression of GrB (32/45, 71.1%) and perforin (31/42, 73.8%) was noticed. CD30 was expressed, at least partially, in 40.6% (13/32) of the cases. Ki-67 proliferation index varied from 50% to 90%, with an average of 72.7%. In all cases, the tumor cells expressed neither ALK1 nor CD20. (Fig 2)

Positive ISH signals of EBER were identified in tumor cells in all cases. Of the 17 tested cases, 9 (52.9%) demonstrated monoclonal TCR gene rearrangement, with a phenotype of TCR γδ+, αβ+ and αβ/γδ+ in 3 patients each.

### Treatment and outcome

Forty-two patients received intestinal segment resection. A total of 35 patients (63.6%) were treated with systemic chemotherapy. Another one (1.8%) received chemoradiotherapy and subsequent autologous hematopoietic stem cell transplantation. Eleven (20.0%) failed to receive any intervention due to poor health condition. And no information was available for the remaining 8 (14.6%) patients. The chemotherapy regimens varied considerably, whereas a DICE regimen (dexamethasone, ifosfamide, cisplatin and etoposide) or L-asparaginase/peg-asparginase-containing regimens, such as SMILE (dexamethasone, methotrexate, ifosfamide, L-asparaginase and etoposide) and P-GEMOX (peg-asparginase, gemcitabine and oxaliplatin), were most frequently employed. Other regimens including the CHOP (cyclophosphamide, doxorubicin, vincristine and prednisone), CVAD (cyclophosphamide, vincristine, doxorubicin and dexamethasone), MINE (mesna, ifosfamide, mitoxantrone and etoposide) and EPOCH (etoposide, prednisone, vincristine, cyclophosphamide and doxorubicin) were variably adopted.

**Fig 3:**
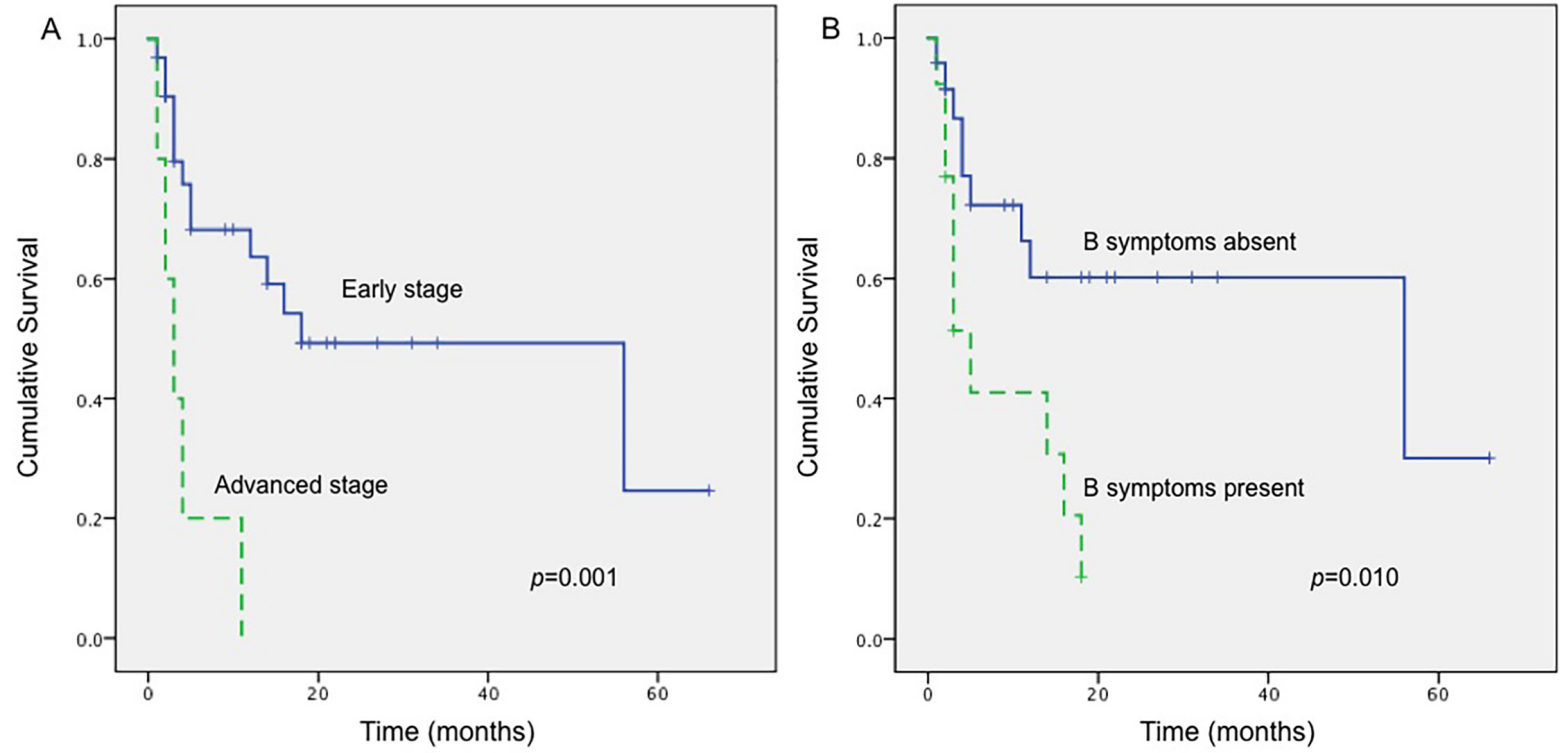
Kaplan-Meier survival curves of patients with PI-ENKTCL. (A) Patients with early stage disease had superior survival compared with those with advanced stage disease (*p* = 0.001). (B) Patients with B symptoms were associated with a much poorer survival than those without B symptoms (*p* = 0.010).

The follow-up information was available in 37 patients with a follow-up time averaged 12.7 months (range, 1–66 months). Three cases relapsed 16–20 months after diagnosis, 19 patients died of the disease and 18 were alive at the last contact. Kaplan-Meier analysis revealed that the stage of disease at diagnosis and B symptoms were statistically associated with the OS (*p* = 0.001, *p* = 0.010, respectively) (Fig 3). Patients at earlier stage and those without B symptoms had relatively longer OS. Further multivariate analysis confirmed that both the stage and B symptoms were independent prognostic indicators. In addition, the prognosis of patients with intestinal perforation or old age (≧40 years) tended to be less favorable. Interestingly, patients with a T-lineaged tumor tended to have a better survival. Nevertheless, none of the above-mentioned findings could be proven statistical significant (*p*>0.05). Other parameters, including the tumor location, size of tumor cells, CD30 or CD56 positivity and Ki-67 index, did not show any correlation with the prognosis.

### A distinct subgroup of intestinal CD4-positive ENKTCL

**Fig 4:**
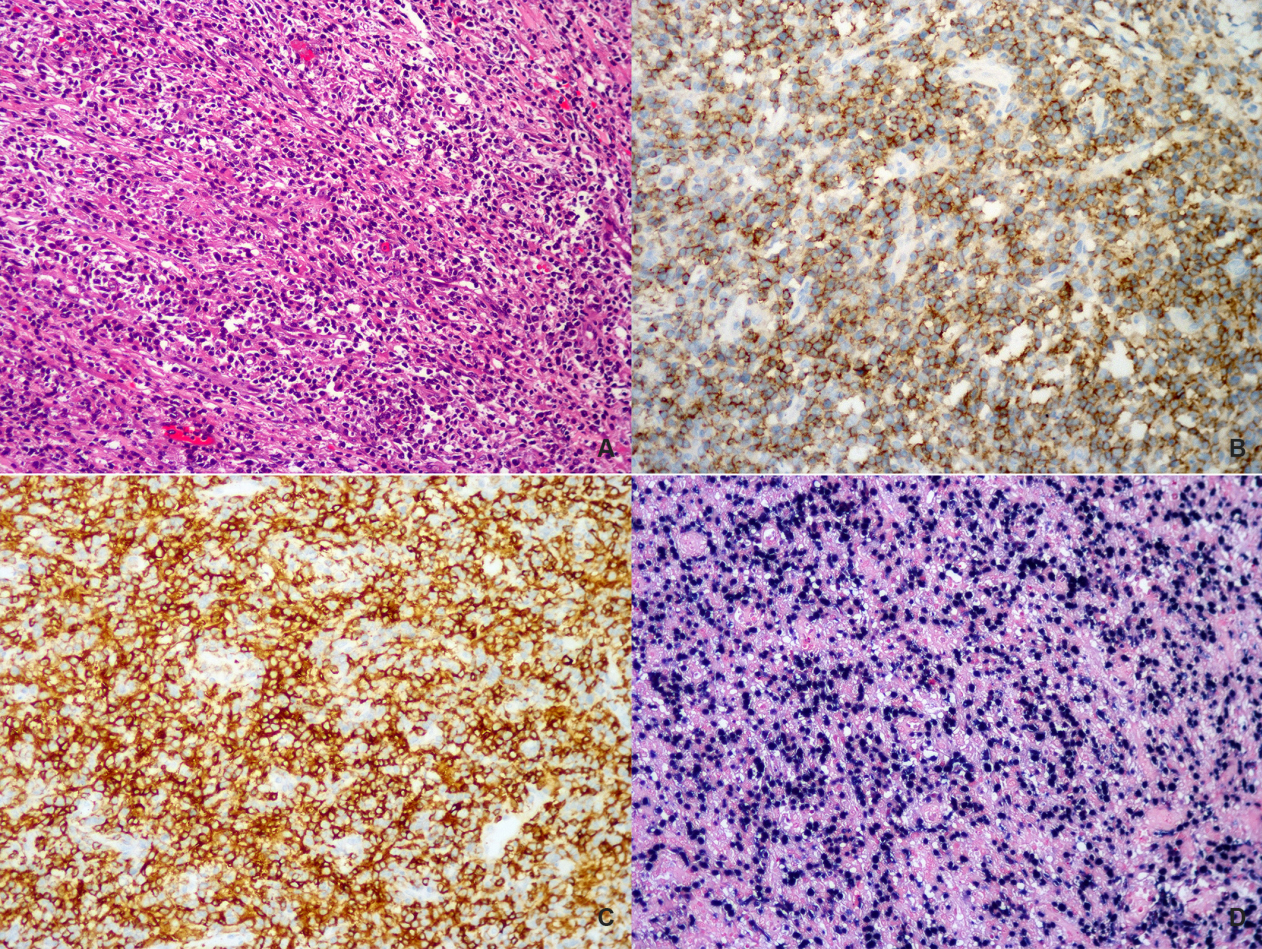
A representative case of primary intestinal CD4+ ENKTCL involving the ileum. (A) The neoplastic cells were predominantly small to medium-sized ones with slightly irregular nuclei. Tumor cells were diffusely positive for (B) CD56 and (C) CD4. (D) In situ hybridization for EBER demonstrated positive signals.

**Fig 5:**
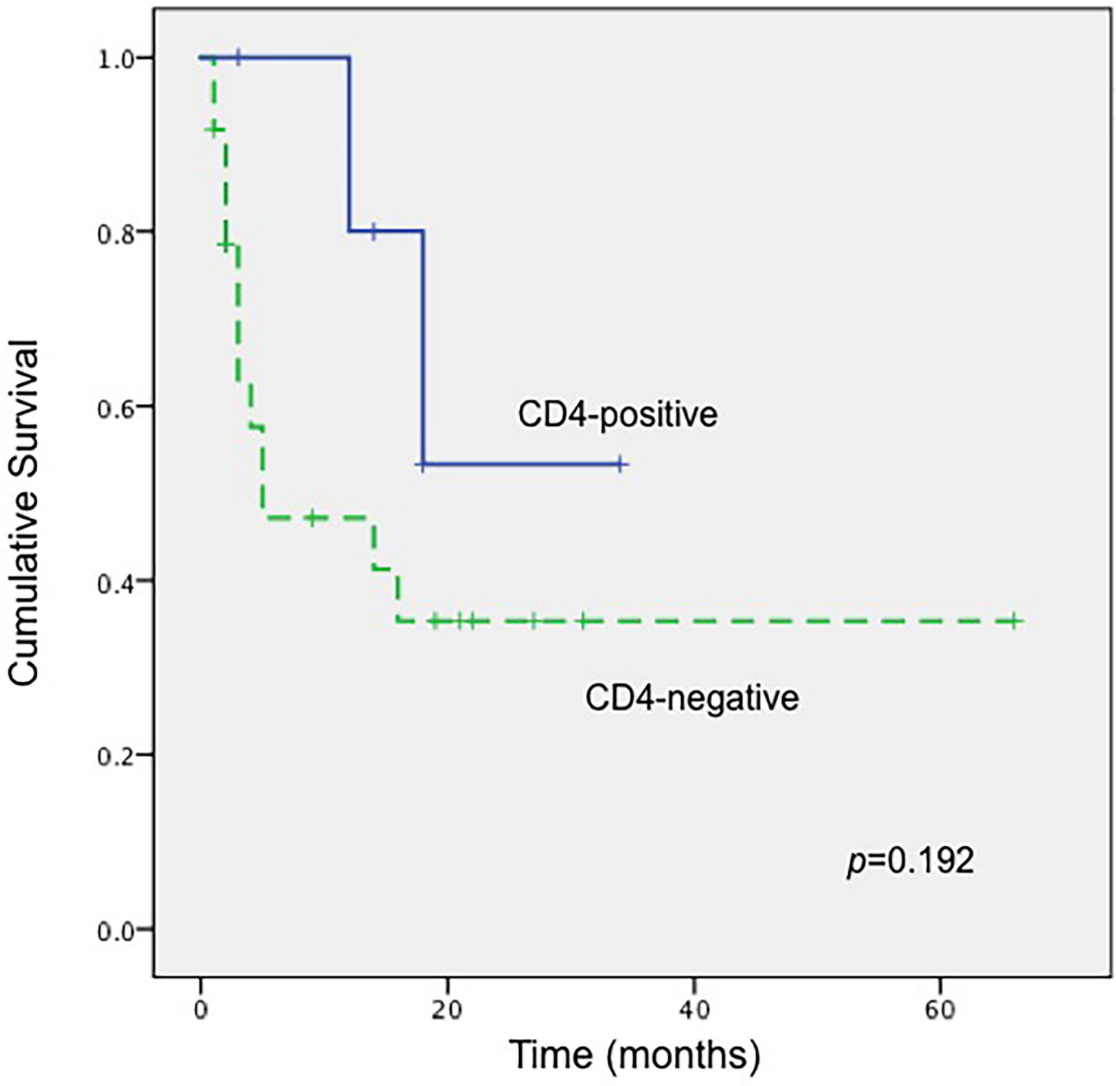
Kaplan-Meier survival curves of patients with PI-ENKTCL according to CD4 expression. Patients with a CD4-positive tumor had a slightly better OS compared with those with CD4-negative ones, although the difference was not statistically significant (*p* = 0.192).

Worthy to be noticed, 6 tumors in the current series demonstrated a CD4-positive phenotype, characterized by a diffuse and intensive positive staining for this marker in almost all the tumor cells (Table 1, Fig 4). All tumors arose from the small intestine with one exception that involved the ileocecal region. Four patients were male and 2 were female, with a median age of 58 years (range, 37–65 years). Morphologically, these tumors were composed predominantly of a proliferation of small to medium-sized atypical lymphoid cells with mild pleomorphism. Large cells were rarely seen, usually accounting for less than 10% of the tumor cell population. All the cases were positive for CD56, cytotoxic molecules and EBER, and clonal TCR gene rearrangements were demonstrated in all tested cases. Two patients died of disease at 18 and 12 months after diagnosis, respectively, and the remaining 4 were alive at the last contact. The one-year OS of these CD4-positive cases was 80.0%, better than that of CD4-negative ones (47.1%). Kaplan-Meier analysis revealed a superior OS of these CD4-positive ENKTCL cases compared with those CD4-negative ones, although the difference did not reach statistical significance (*p* = 0.192, Fig 5).

**Table 1:** The clinicopathological features of primary intestinal CD4+ ENKTCL.

Case No.	Sex/Age (years)	Site	Stage	Status	Follow-up time (months)	Tumor cell size	CD4	CD8	CD56	Ki-67 (%)	EBER	TCR gene rearrangement	Treatment
1	Female/37	Ileum	IIE	Alive	3	Small	+	-	+	70	+	Monoclonal	Resection +chemotherapy
2	Male/62	Ileum	IE	Alive	14	Medium	+	-	+	60	+	Monoclonal	Resection +chemotherapy
3	Male/55	Small intestine	IE	Alive	18	Small/medium	+	-	+	80	+	ND	Resection +chemotherapy
4	Male/65	Small intestine	IE	Dead	12	Small/medium, scattered large cells	+	-	+	60	+	Monoclonal	Resection*
5	Male/60	Duodenum	IE	Alive	34	Small/medium, scattered large cells	+	-	+	80	+	Monoclonal	Chemotherapy
6	Female/39	ileocecal region	IIE	Dead	18	Small/medium, scattered large cells	+	-	+	70	+	ND	Resection+chemotherapy

ND, not done.

*, information on subsequent treatment of this patient is unavailable.

## Discussion

The GI tract is one of the most common extranodal sites that can be involved by lymphomas [12]. However, ENKTCL originating from the intestine is extremely rare. So far only few studies with limited number of cases were documented in the English literature [13, 14]. Due to its rarity, the clinical and pathological features of PI-ENKTCL are less well defined. We thus collected a large cohort of cases to observe the clinicopathological characteristics of this uncommon disease. To the best of our knowledge, the current data set may represent by far the largest one focusing on intestinal ENKTCL.

Based on our findings, PI-ENKTCL more frequently affected middle-aged adults in their forties with a male predominance, which is in consistent with several previous studies [12, 15]. Regarding the sites of involvement, small intestine is most commonly affected, followed by colon and the ileocecal junction. This result is in good concordance with the findings by Kim et al [16], but differs with some other studies, which demonstrated that large intestine is much more commonly involved by this type of lymphoma [12, 17]. The most common presenting symptom is abdominal pain, with or without fever, as same as Fang et al indicated before [13]. Endoscopically, most intestinal ENKTCL presents as ulcerative lesions, being prone to perforation, rather than a polypoid mass, suggesting a potential more aggressive behavior of this tumor [12].

The variety of histologic appearance of intestinal ENKTCL might have reflected the striking heterogeneity of this tumor. In terms of immunophenotype, CD56 appears to be a useful marker aiding in the diagnosis, with an expression rate varying between 60% and 100% in different series [3, 8, 18]. It has been proposed that CD56 might be expressed more frequently in NK-derived tumors than T-cell-originated ones [4]. Our results, together with those of some other studies, however, did not reveal such a difference [3, 8, 18]. The expression of one or more cytotoxic molecules, including TIA-1, GrB and perforin, is invariably present in every case, with TIA-1 expression being more frequently detected [4, 18]. As a marker related to cell activation, CD30 was frequently expressed by the tumor cells in the current series, which presumably reflected the fact that some ENKTCL cases feature an activated phenotype. The relatively high positivity for CD30 in intestinal ENKTCL is also in good keeping with that a higher percentage of CD30 expression was noticed in extra-nasal ENKTCL lesions than those nasal ones [4, 19].

ENKTCL is almost constantly associated with EBV, which is suspected to play an important role in the oncogenesis of this disease [20]. Identification of EBV-encoded EBER is therefore essential for the establishment of a diagnosis of ENKTCL. In the current series, nearly half of the intestinal ENKTCL cases that submitted for TCR gene rearrangement detection demonstrated a germline configuration of the TCR genes, confirming a genuine NK-cell origin of the tumor, however, T-cell-derived tumors accounted for 52.9% of all tested cases, an incidence slightly higher than those previously reported ranging from 0%-46% [4, 5, 10, 14, 19, 21–23]. The clinical significance of cell origin of an intestinal ENKTCL remains largely unknown, although in general, ENKTCL cases of different lineage do not show distinct clinopathological features including the survival [4, 10, 19]. We noticed a trend of a better survival in patients with T-cell-derived intestinal ENKTCLs than those with NK-lineaged ones, although the survival advantage was not statistically significant. Pongpruttipan et al seemed to have similar findings although the tumors in their series were mostly located in the upper aerodigestive tract instead of the intestine [4]. Chuang and colleagues also noticed a superior survival of those EBV-related cytotoxic T-cell lymphomas arising in the intestine compared with their NK-cell counterparts, but they referred to the former as “NK-like peripheral T-cell lymphomas”, possibly an entity distinct from the genuine PI-ENKTCLs or enteropathy-associated T-cell lymphomas (EATL) [24].

Intestinal ENKTCL must be distinguished from its morphological mimics. It is not uncommon that some intestinal ENKTCL cases are misdiagnosed as peripheral T-cell lymphoma, not otherwise specified (PTCL, NOS) due to CD3 positivity and a high proliferation rate of the tumor cells. However, the later usually lacks the characteristic massive necrosis and angiodestructive growth pattern. In general, EBER is absent in PTCL, NOS. CD56 expression is also less commonly seen in this tumor compared with ENKTCL. Type II EATL might be confused with intestinal ENKTCL due to their common CD56+ and cytotoxic phenotype [4]. Nevertheless, the former is composed of monotonous medium-sized tumor cells with marked epitheliotropism. Necrosis is uncommon in type II EATL, too. The tumor cells typically co-express CD8 but lack EBER [25], although it still remains controversial that whether some EATL-appearing but EBER-positive intestinal T-cell lymphomas can be labeled as an ENKTCL. Occasionally, PI-ENKTCLs consisting mainly of large or anaplastic cells may show CD30 positivity and thus, might be potentially misinterpreted as anaplastic large cell lymphoma (ALCL) or the classical form of EATL. Detection for EBER is critical for the distinction between these diseases. Rare examples of indolent T- or NK-cell lymphoproliferative disorders of the GI tract, such as the NK-cell lymphomatoid gastroenteropathy and indolent T-cell lymphoproliferative disorder of the GI tract, may morphologicaly or phenotypically mimic ENKTCL, too [26, 27]. These lesions, however, differ from ENKTCL by their characteristic indolent clinical course and an absence of EBV in the lesional cells.

Given the rarity of PI-ENKTCL and its clinicohistological heterogeneity, there is currently no consensus approached on the proper treatment strategy [16, 28]. A timely surgical operation might be necessary since it has been found that patients receiving surgery before perforation usually feature an improved outcome compared to those undergoing surgery later or without surgery [12]. Moreover, surgery with subsequent chemotherapy might be more beneficial than initially treatment with chemotherapy for patients eligible for surgery [16]. Nevertheless, Fang et al presumed that operation might accelerate deterioration of the disease [13]. On the other hand, so far there is no standard chemotherapeutic regimen for ENKTCL [29]. It has been reported that patients with GI ENKTCL who received nonanthracycline-based or intensified regimens did not show a significant survival difference compared with those who received CHOP or CHOP-like regimens regardless of surgery [16]. Recently, the efficacy of L-asparaginase or peg-asparginase in the treatment of ENKTCL has been confirmed, and clinical trials using L-asparaginase or peg-asparginase-based chemotherapeutic regimens have achieved promising results [29]. Yet, about half of our patients died during the follow-up period irrespective of the administration of comprehensive treatment. Novel effective approaches are thus warranted to improve the survival of patients with this rare but fatal disease.

Both our study and some previous ones had demonstrated a large proportion of patients with intestinal ENKTCL presented with early stage diseases [13], these patients, however, tended to exhibit highly aggressive behavior and thus, conferred an inferior prognosis as compared to their nasal counterparts [16]. For such a contradiction, one reasonable explanation lies that the overt extra-nasal tumors in some patients might represent actually the extra-nasal dissemination of an occult nasal lesion. And the primary nasal lesions are easily to be missed or overlooked only because of their small sizes [3]. Therefore, a PET-CT scan, nasal panendoscopy and careful imaging of the aerodigestive tract, with biopsy when necessary, are mandatory for each new case of ENKTCL, irrespective of the initial presentation sites, as indicated previously [1, 3, 30]. However, all cases enrolled in the current study have been submitted for a precise staging work-up and hence secondary onset can be excluded. Thus there might be some other reasons accounting for the poor outcome of PI-ENKTCL. It has been suggested that old age, intestinal perforations, B symptoms are independent prognostic factors indicating a poor outcome of patients with GI-ENKTCL, and surgery prior to the perforation is another key factor that may influence the survival [12]. In our study, the results have confirmed that both disease stage and B symptoms are significant independent prognostic factors for this rare type of tumor. The prognostic significance concerning CD56 expression in ENKTCL is controversial [31, 32]. We have failed to find any correlations between CD56 expression and OS, probably due to the limited number of CD56-negative cases.

Of interest, we noticed and described for the first time 6 cases of intestinal T-cell lymphomas characterized by their predilection for small intestine involvement, predominance of small to medium-sized tumor cells, a CD4+, CD56+, EBER+ T-cell phenotype and importantly, relatively favorable outcome. We tentatively labeled these lesions as ENKTCL mainly based on their cytotoxic T-cell phenotype and EBV positivity. CD4-positive ENKTCL is extremely rare. In a large cohort of 67 cases with ENKTCL, Pongpruttipan et al identified only one CD4-positive case, which might be of αβ T-cell origin [4]. We suppose these peculiar CD4+, CD56+, EBER+ tumors of the small intestine may represent a special variant of ENKTCL or even novel entity due to their unique clinical and pathological features. Recognition of these CD4-positive tumors may aid in predicting the prognosis and tailoring a personalized treatment strategy to the patients. Further work is needed to elucidate the biological nature of the disease and its relationship with the classical form of intestinal ENKTCL.

In conclusion, PI-ENKTCL is a rare condition. The disease predominantly affects middle-aged males and features frequently aggressive clinical course and poor outcome. Independent inferior prognostic indicators may include the stage of disease and B symptoms. A subgroup of cases with a CD4-positive phenotype might represent a unique variant or clinicopathological entity. Further molecular and clinical studies are encouraged for the better characterization and management of the disease.

## Supporting Information

S1 Table The clinicopathological characteristics of patients with primary intestinal extranodal natural killer/T-cell lymphoma, nasal type. (XLSX)
